# Extranodal Natural Killer/T-Cell Lymphoma: A Rare Nasal-Type Case

**DOI:** 10.4274/tjh.2015.0169

**Published:** 2016-02-17

**Authors:** Esra Sarıbacak Can, Harika Okutan, Murat Albayrak, Ünsal Han

**Affiliations:** 1 Dışkapı Yıldırım Beyazıt Research and Training Hospital, Clinic of Hematology, Ankara, Turkey; 2 Dışkapı Yıldırım Beyazıt Research and Training Hospital, Clinic of Pathology, Ankara, Turkey

**Keywords:** Extranodal natural killer/T-cell lymphoma, non-Hodgkin lymphoma

## TO THE EDITOR

Nasal type extranodal natural killer (NK) NK-cell/T-cell lymphoma (NKTCL) is a rare extranodal lymphoma of NK-cell or T-cell origin that most commonly affects immunocompetent middle-aged men of Asian or Native American descent [1]. The pathogenesis is not understood completely, but it is related in part to infection of the tumor cells with Epstein-Barr virus (EBV) [2]. Around 6-7% of all non-Hodgkin’s lymphoma (NHL) in Southeast Asia accounts for NKTCL. However, the incidence of NHL is lower in the United States at 1.5% [3,4].

Disease within the nasal cavity has a better prognosis. Radiation therapy alone can be curative. Over 60% of patients with stage 1 disease remain in long-term remission following treatment with radiation therapy with or without chemotherapy [5]. Nasal disease may be cured with radiotherapy at a rate of 85%. However, the relapse rate is high at 25%. Therefore, it is highly crucial for this aggressive disease to be diagnosed and treated at an early stage [6,7].

**Figure 1:**
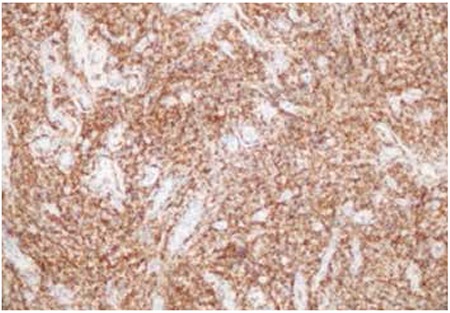
Strong staining of diffuse cytoplasmic natural killer cells with CD56 (CD56, IHC, 200x).

In our case, a 29-year-old female complained of nasal obstruction and had a necrotizing mass in the upper middle concha at the right nasal cavity; biopsy was planned. CD56 was strongly positive (Figure 1), CD8 was positive in a few scattered cells, and CD4 was positive in the majority of infiltrating T cells in the sample of necrotic tissue pieces. TIA-1 and perforin were commonly positive. Granzyme was commonly strongly cytoplasmic-positive. Epstein-Barr encoding region (EBER) in situ hybridization analysis was done with a probe cocktail containing EBV. Early RNA transcript showed that NKTCL compatibility existed with commonly strongly nuclear-positivity in EBER infiltrating cells. Positron emission tomography-computed tomography (PET-CT) revealed pathologically heterogeneous soft tissue mucosal thickening, pushing the nasal septum slightly to the left and hypertrophy of the right ethmoid cells in the upper middle concha level of the nasal cavity, with increased metabolic uptake (SUVmax: 3.18). The patient was diagnosed with stage 1E based on PET-CT evaluation and received a total of 38 Gy external radiotherapy at 200 cGy daily. No involvement was detected month after radiotherapy and complete response was considered to have occurred after 1 year.

NKTCL of the palate and sinuses has been reported in many cases. However, the incidence of NKTCL is much lower in the United States. Nasal obstruction, bleeding, pain, or local swelling are usually observed and ulcerative, destructive lesions within extranodal sites can be produced. Often it is associated with EBV. Immunophenotypically, the tumor cells express CD2, CD3, and CD56. The cells can lack CD56 and express CD8+ T-cell antigens in some cases [7]. The course of NKTCL, nasal type, is aggressive where a 5-year overall survival ranges from 25% to 50% [8].

Symptoms of nasal type NKTCL can include nasal discharge, nasal obstruction and other nonspecific sinonasal symptoms. However, sore throat and dysphagia, also known as symptoms of nasal type NKTCL, are frequently missed and treated as viral and bacterial pharyngitis, which leads to late diagnosis. Therefore, morbidity and mortality are increased. NK/T-cell lymphoma, nasal type, is rarely observed in Turkey and early diagnosis of the disease is of vital importance.
